# 19.2 RECOVERY THROUGH RELOCATION: FROM NURSING HOME TO COMMUNITY USING COGNITIVE ADAPTATION TRAINING

**DOI:** 10.1093/schbul/sby014.076

**Published:** 2018-04-01

**Authors:** Natalie Maples

**Affiliations:** University of Texas Health Science Center at San Antonio

## Abstract

**Background:**

Texans with severe mental illness live 29 years less than other Americans and have more health problems earlier in life. Since 2001, over 46,000 Texans have returned home under the State’s Money Follows the Person program and federal demonstration grant. Despite this impressive achievement, people with mental health and substance use conditions continue to be institutionalized in nursing facilities (NF). Nationally, the number of NF residents under age 65 with a primary diagnosis of mental illness is nearly three times that of older residents. The Texas Money Follows the Person Behavioral Health Pilot (MFP-BHP), assists nursing facility residents with co-morbid mental and physical illnesses relocate into the community. The transition from institutionalization to independent living is a crucial time for treatment intervention to maintain independence and reduce high risk adverse outcomes, including hospitalization, exacerbation of symptoms or homelessness.

**Methods:**

In addition to service coordination from Managed Care Organizations, participants receive Cognitive Adaptation Training (CAT) for six months in the nursing facility and one year in the community. CAT is a home-based psychosocial treatment utilizing environmental supports such as medication containers, signs, checklists and the organization of belongings to bypass deficits in cognitive functioning and cue and sequence adaptive behavior, to improve functional outcomes for individuals with mental illness. This demonstration project assessed the effectiveness of providing CAT to improve functional and social outcomes, measured at baseline, each three months for one year, and each six months post intervention for one additional year.

**Results:**

Over 500 individuals have been transitioned into the community since 2008, with 60% maintaining independence. Findings indicate a significant improvement in targeted functional outcomes post facility discharge on the Multnomah Community Ability Scale (p<.0001), Social and Occupational Functioning Scale (p<.001) and the Quality of Life Scale (p< .01). Preliminarily analyses indicate that Medicaid costs for participants are considerably lower on average than costs prior to discharge. At the end of 2015, the savings to the state via the Pilot were tens of millions. The Pilot ends in December 2017 and final cost analyses will be conducted at this time.

**Discussion:**

CAT was successfully applied to persons with co-occurring mental and physical disorders relocating from nursing facilities to independent living environments with good preliminary outcomes indicating better quality of life, social and occupational role function and in overall community functioning. The majority of persons have successfully remained in the community. The MFP Behavioral Health Pilot shows Medicaid participants residing in nursing facilities with significant mental health issues can successfully maintain their residence in the community which results in significant cost savings, even taking into account the standard MFP costs plus the intervention. MFP Pilot participants improved their functional status, which extended after the intervention period ended. Current implementation efforts are in place to integrate and sustain CAT in the statewide managed care system.

